# Crystal structure of the ‘missing’ inter­metallic compound BaCu_5_

**DOI:** 10.1107/S2056989026008248

**Published:** 2026-08-14

**Authors:** Kiran Avinash, Justin Andrews

**Affiliations:** a Department of Chemistry, Purdue University, 560 Oval Dr., West Lafayette, IN 47907-2084, USA; University of Aberdeen, United Kingdom

**Keywords:** crystal structure, inter­metallic, electride, missing phase

## Abstract

The copper-rich inter­metallic compounds CaCu_5_ and SrCu_5_ are isostructural but the isomorphous compound BaCu_5_ (barium penta­copper) has not been reported. The absence of any compound with the composition of BaCu_5_ in the Ba–Cu phase diagram has previously been attributed to much larger radius of Ba. The structure of BaCu_5_, which crystallizes in an ortho­rhom­bic cell (space group *Pnma*) with the rare SrZn_5_ structure type is now reported The structure is characterized by a zigzag arrangement of infinite Ba chains ensconced in a Cu matrix. The Ba atom and three Cu atoms lie on special positions with *m* site symmetry and one Cu atom lies on a general crystallographic position.

## Abstract 2

The previously unreported inter­metallic compound BaCu_5_ crystallizes in the rare SrZn_5_ structure type, completing a missing composition in the alkaline-earth copper family.

## 1. Chemical context

**Figure 1:**
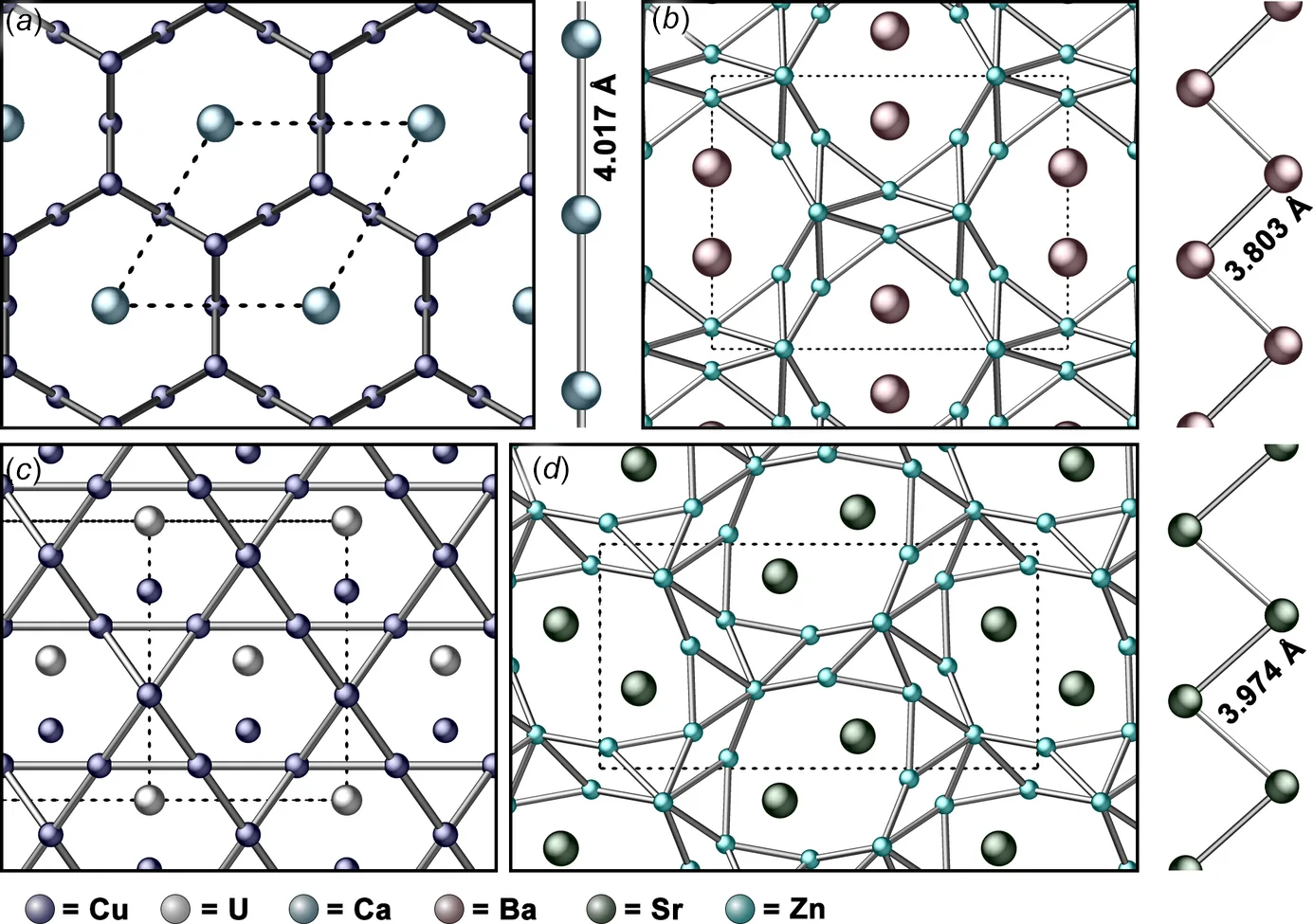
(*a*) Reported structure of CaCu_5_. (*b*) Reported structure of BaZn_5_. (*c*) Reported structure of UCu_5_. (*d*) Reported structure of SrZn_5_. The corresponding arrangement and bonding of alkaline-earth metals in each structure is shown, and relevant bond distances are highlighted. Note that in the case of the structure of UCu_5_, there is no U—U bonding.

Compounds with the stoichiometry (AE/RE)(TM)_5_ (AE/RE = alkaline earth metal or rare earth; TM = transition metal) most often crystallize in the CaCu_5_ structure type (space group *P6/mmm*). Materials that adopt this structure are important as permanent magnets and have been investigated as hydrogen storage alloys (Hou *et al.*, 2007 ▸; Xu *et al.*, 2022 ▸; Zhou *et al.*, 2023 ▸; Liang *et al.*, 2001 ▸). While the CaCu_5_ structure type (*e.g.*, SrCu_5_, YCu_5_, Figure *1a*) is most common, some (AE/RE)(TM)_5_ compounds adopt the BaZn_5_ structure type (space group *Cmcm*) (Fig. 1 ▸*b*). Conversely, UCu_5_ adopts the AuBe_5_ structure type (space group *F*
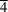
*3m*; Nakamura *et al.*, 1990 ▸) (Fig. 1 ▸*c*). Generally, the connectivity of the Cu metal–metal bonded cage is dictated by the size of the alkaline earth or rare-earth metal. A less-common structure type is the SrZn_5_ structure (space group *Pnma*) (Fig. 1 ▸*d*), for which only four members have been reported (Simura & Yamane, 2019 ▸; Schwickert & Pöttgen, 2014 ▸; Bruzzone & Merlo, 1983 ▸). The Cu cage arrangements for each structure are shown in Fig. 1 ▸*a*–*d*.

For the (AE)Cu_5_ series of materials, the eponymous CaCu_5_ and the larger SrCu_5_ compounds are known to adopt the same structure (Bruzzone, 1971 ▸; Boeije *et al.*, 2017 ▸). However, a compound with the composition of BaCu_5_ has never been reported. The original study that systematically explored the (Ca/Sr/Ba)—Cu phase diagram remarked on the conspicuous absence of a BaCu_5_ compound and attributed its absence to the much larger size of Ba (Bruzzone, 1971 ▸).

Here we report on the structure of BaCu_5_ and show that unlike CaCu_5_, SrCu_5_, and BaZn_5_ (Bruzzone *et al.*, 1985 ▸) it adopts the very rare SrZn_5_ structure type (Bruzzone & Merlo, 1983 ▸).

## 2. Structural commentary

**Figure 2:**
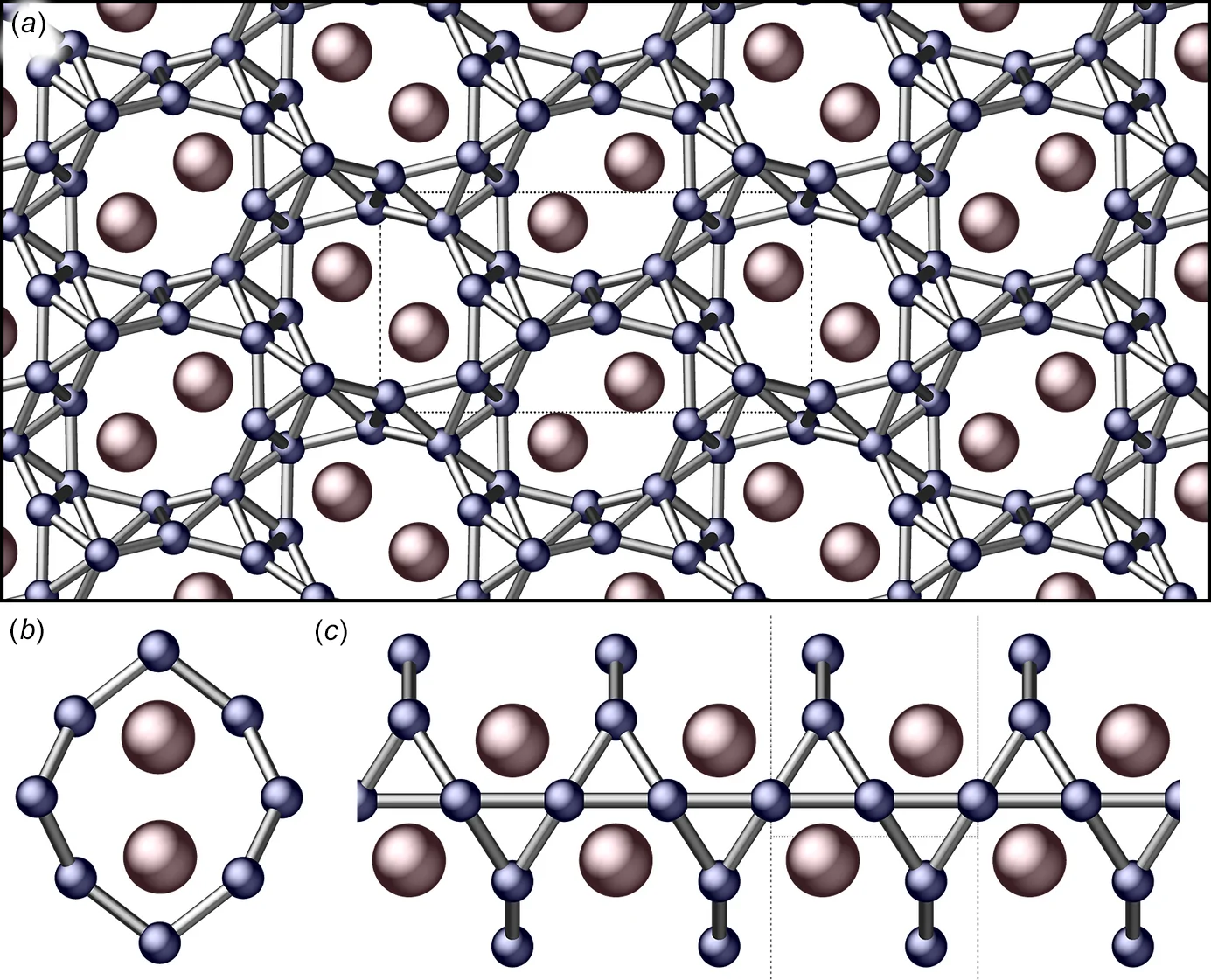
Structure of BaCu_5_. (*a*) projection of the structure along the crystallographic *b*-axis direction. (*b*) Ba occupies one-dimensional tunnels in the Cu framework. (*c*) View of one zigzag chain of Ba atoms propagating along the crystallographic *a-*axis direction.

BaCu_5_ crystallizes in the ortho­rhom­bic *Pnma* space group and adopts the rare SrZn_5_ structure type, rather than the more common CaCu_5_ structure type adopted by the lighter alkaline-earth analogues CaCu_5_ and SrCu_5_. The asymmetric unit contains one crystallographically independent Ba atom and four crystallographically independent Cu atoms. The Ba atom and three Cu atoms occupy special positions with site symmetry *m*, whereas one Cu atom occupies a general position. The structure consists of a three-dimensional Cu framework that forms one-dimensional tunnels extending parallel to the crystallographic *a* axis, within which infinite zigzag chains of Ba atoms are accommodated (Fig. 2 ▸).

The Ba atoms form planar zigzag chains with a nearest-neighbor Ba—Ba separation of 3.8366 (5) Å. The geometry of the chain is defined by a Ba—Ba—Ba angle (φ) of 81.70 (2)° and a Ba—Ba—Ba—Ba dihedral angle (ω) of 180°, indicating that the chains are coplanar. The corresponding zigzag angle in the isostructural low-temperature SrZn_5_ phase is slightly larger (φ = 83.05°), while the overall chain topology is retained.

**Figure 3:**
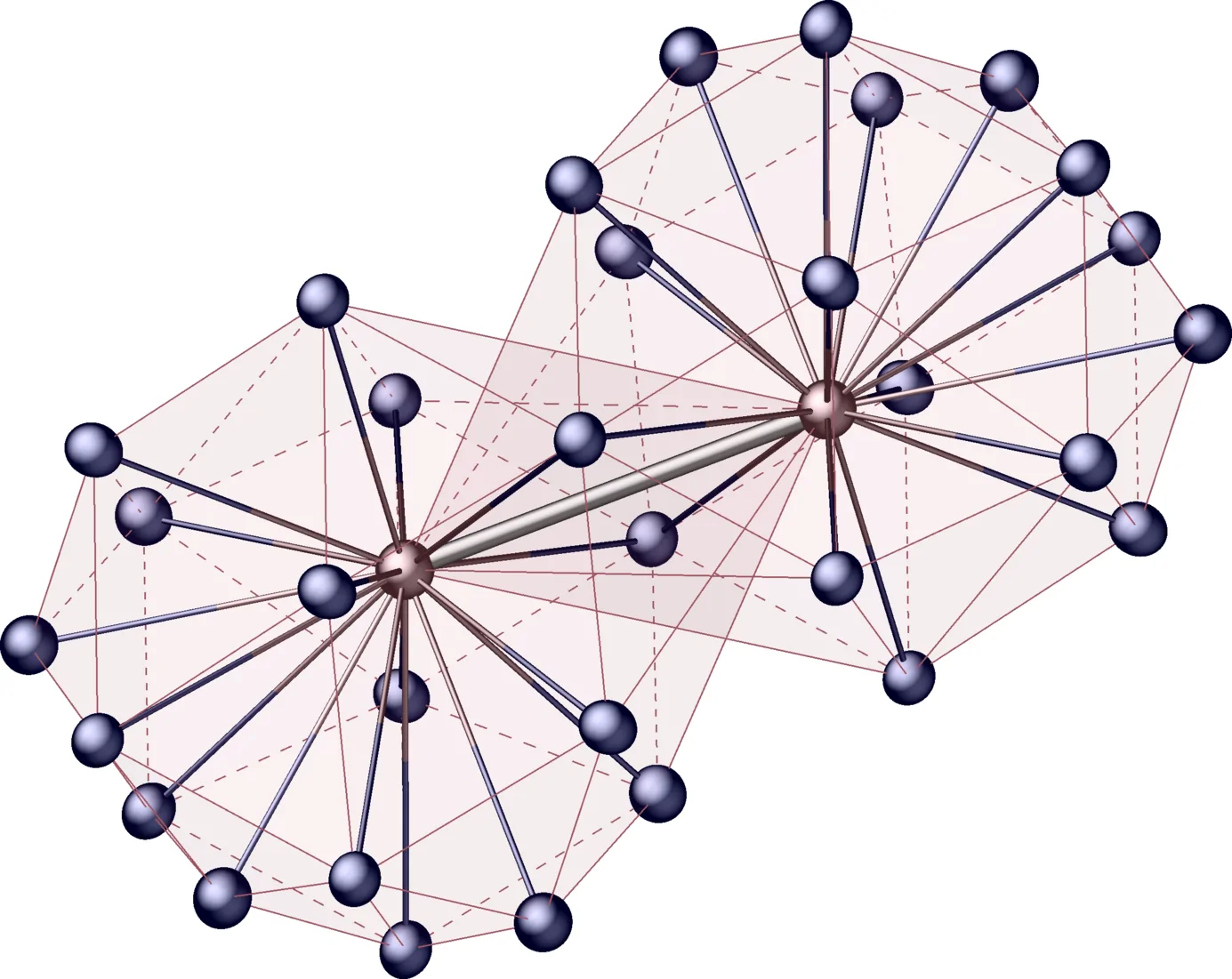
Local coordination around the Ba atoms in the structure. Each Ba atom is bonded to two neighboring Ba atoms and 17 Cu atoms with an average bond distance of 3.398 Å. The Ba—Ba distance is 3.8366 (5) Å, which is slightly longer than the Ba—Ba distance in BaZn_5_ and slightly shorter than the Sr—Sr distance in SrZn_5_.

Each Ba atom in BaCu_5_ is coordinated by 17 Cu atoms with an average Ba—Cu distance of 3.3966 (6) Å (Fig. 3 ▸). The Ba—Ba distance is slightly longer than that reported for BaZn_5_ (3.803 Å) and slightly shorter than the corresponding Sr—Sr distance (3.974 Å) in the isostructural SrZn_5_ phase. The Cu framework in BaCu_5_ is constructed from four crystallographically distinct Cu sites. The shortest Cu—Cu contacts are found around the general position Cu site, with distances ranging from 2.5012 (9) to 2.5404 (10) Å, whereas longer Cu—Ba contacts occur between 3.5163 (7) and 3.6461 (8) Å.

## 3. Database survey

A search of the PDF-5+ database (Kabekkodu *et al.*, 2024 ▸) for the CaCu_5_ structure type returned 2080 entries: 434 of them contained Cu as the transition metal and 11 contained Ba as the alkaline earth metal but no entries contained both Ba and Cu. In contrast, a search for the SrZn_5_ structure type returned just four hits, one of them being SrZn_5_ itself, highlighting the exceptional rarity of this structure type

## 4. Synthesis and crystallization

The title compound was synthesized by modification of a previously reported synthesis (Wan *et al.*, 2024 ▸). Ba beads (Sigma Aldrich 99%) and Cu powder (Thermo Fisher 99%, 325 mesh) were pressed together in a 1:1 stoichiometric ratio in an argon filled glove box. The solids were sealed in a quartz ampoule under vacuum and heated at 873 K for 12 h. The reaction was then cooled down to room temperature over the course of 6 h. After the reaction, a mixture of several phases was present in the melt including single crystals of the layered electride BaCu and several previously unreported and highly disordered barium suboxides (Ba_7_O_3_) resulting from reaction between Ba and the quartz ampoule. Several unsuccessful attempts were made to synthesize phase-pure samples of BaCu_5_ directly in quartz, steel, and niobium crucibles. Single crystals of BaCu_5_ were picked out from the melt of the reaction. The broken pieces of the melts were submerged in polybutene oil inside an argon filled glovebox. The crystals were quickly transferred to the goniometer head.

## 5. Refinement

Crystal data, data collection and structure refinement details are summarized in Table 1 ▸.

## Supplementary Material

Crystal structure: contains datablock(s) I. DOI: 10.1107/S2056989026008248/hb8240sup1.cif

Structure factors: contains datablock(s) I. DOI: 10.1107/S2056989026008248/hb8240Isup2.hkl

CCDC reference: 2579957

Additional supporting information:  crystallographic information; 3D view; checkCIF report

## Figures and Tables

**Table 1:** Experimental details

Crystal data	
Chemical formula	BaCu_5_
*M*_r_	455.04
Crystal system, space group	Orthorhombic, *Pnma*
Temperature (K)	150
*a*, *b*, *c* (Å)	12.8420 (7), 5.0189 (3), 6.5445 (6)
*V* (Å^3^)	421.81 (5)
*Z*	4
Radiation type	Mo *K*α
μ (mm^−1^)	33.71
Crystal size (mm)	0.13 × 0.08 × 0.02

Data collection	
Diffractometer	Bruker AXS D8 Quest
Absorption correction	Multi-scan (*SADABS*; Krause *et al.*, 2015 ▸)
*T*_min_, *T*_max_	0.135, 0.274
No. of measured, independent and observed [*I* > 2σ(*I*)] reflections	24524, 1112, 877
*R*_int_	0.080
(sin θ/λ)_max_ (Å^−1^)	0.834

Refinement	
*R*[*F*^2^ > 2σ(*F*^2^)], *wR*(*F*^2^), *S*	0.030, 0.059, 1.09
No. of reflections	1112
No. of parameters	34
Δρ_max_, Δρ_min_ (e Å^−3^)	1.69, −1.76

Computer programs: *APEX5* and *SAINT* (Bruker, 2025 ▸), *SHELXT* (Sheldrick, 2015*a* ▸), *SHELXL2025/1* (Sheldrick, 2015*b* ▸) and *ShelXle* (Hübschle *et al.*, 2011 ▸).

## supplementary crystallographic information

### Barium pentacopper Crystal data

**Table:**

BaCu_5_	*D*_x_ = 7.165 Mg m^−^^3^
*M**_r_* = 455.04	Mo *K*α radiation, λ = 0.71073 Å
Orthorhombic, *P**n**m**a*	Cell parameters from 6137 reflections
*a* = 12.8420 (7) Å	θ = 3.2–35.8°
*b* = 5.0189 (3) Å	µ = 33.71 mm^−^^1^
*c* = 6.5445 (6) Å	*T* = 150 K
*V* = 421.81 (5) Å^3^	Block, black
*Z* = 4	0.13 × 0.08 × 0.02 mm
*F*(000) = 804	

### Barium pentacopper Data collection

**Table:**

Bruker AXS D8 Quest diffractometer	1112 independent reflections
Radiation source: fine focus sealed tube X-ray source	877 reflections with *I* > 2σ(*I*)
Triumph curved graphite crystal monochromator	*R*_int_ = 0.080
Detector resolution: 7.4074 pixels mm^-1^	θ_max_ = 36.4°, θ_min_ = 3.2°
ω and phi scans	*h* = −21→21
Absorption correction: multi-scan (SADABS; Krause *et al.*, 2015)	*k* = −8→8
*T*_min_ = 0.135, *T*_max_ = 0.274	*l* = −10→10
24524 measured reflections	

### Barium pentacopper Refinement

**Table:**

Refinement on *F*^2^	0 restraints
Least-squares matrix: full	Primary atom site location: dual
*R*[*F*^2^ > 2σ(*F*^2^)] = 0.030	Secondary atom site location: difference Fourier map
*wR*(*F*^2^) = 0.059	*w* = 1/[σ^2^(*F*_o_^2^) + (0.0111*P*)^2^ + 7.083*P*] where *P* = (*F*_o_^2^ + 2*F*_c_^2^)/3
*S* = 1.09	(Δ/σ)_max_ < 0.001
1112 reflections	Δρ_max_ = 1.69 e Å^−^^3^
34 parameters	Δρ_min_ = −1.76 e Å^−^^3^

### Barium pentacopper Special details

**Table:**

Geometry. All esds (except the esd in the dihedral angle between two l.s. planes) are estimated using the full covariance matrix. The cell esds are taken into account individually in the estimation of esds in distances, angles and torsion angles; correlations between esds in cell parameters are only used when they are defined by crystal symmetry. An approximate (isotropic) treatment of cell esds is used for estimating esds involving l.s. planes.

### Barium pentacopper Fractional atomic coordinates and isotropic or equivalent isotropic displacement parameters (Å^2^)

**Table:**

	*x*	*y*	*z*	*U*_iso_*/*U*_eq_	
Ba1	0.58888 (3)	0.750000	0.13690 (6)	0.00983 (8)	
Cu1	0.48045 (6)	0.750000	0.57102 (13)	0.00970 (14)	
Cu2	0.35376 (4)	0.49998 (11)	0.35225 (9)	0.00888 (10)	
Cu3	0.28152 (7)	0.750000	0.05359 (12)	0.00979 (15)	
Cu4	0.21455 (6)	0.250000	0.16200 (12)	0.00922 (14)	

### Barium pentacopper Atomic displacement parameters (Å^2^)

**Table:**

	*U*^11^	*U*^22^	*U*^33^	*U*^12^	*U*^13^	*U*^23^
Ba1	0.01055 (14)	0.01004 (15)	0.00890 (14)	0.000	0.00060 (13)	0.000
Cu1	0.0087 (3)	0.0103 (3)	0.0101 (3)	0.000	−0.0008 (3)	0.000
Cu2	0.0100 (2)	0.0077 (2)	0.0089 (2)	−0.00029 (18)	−0.00062 (19)	0.00011 (19)
Cu3	0.0115 (3)	0.0099 (3)	0.0080 (3)	0.000	−0.0009 (3)	0.000
Cu4	0.0098 (3)	0.0099 (3)	0.0080 (3)	0.000	−0.0012 (3)	0.000

### Barium pentacopper Geometric parameters (Å, º)

**Table:**

Ba1—Cu1	3.1640 (10)	Cu1—Cu2^ix^	2.5217 (9)
Ba1—Cu4^i^	3.1935 (9)	Cu1—Cu2^vi^	2.5217 (9)
Ba1—Cu3^ii^	3.1974 (9)	Cu1—Cu4^x^	2.5740 (11)
Ba1—Cu3^iii^	3.2590 (6)	Cu1—Cu1^vi^	2.7228 (7)
Ba1—Cu3^i^	3.2590 (6)	Cu1—Cu1^v^	2.7228 (7)
Ba1—Cu4^iv^	3.2610 (6)	Cu2—Cu3	2.5012 (9)
Ba1—Cu4^ii^	3.2610 (6)	Cu2—Cu2^xi^	2.5092 (11)
Ba1—Cu1^v^	3.2778 (6)	Cu2—Cu2^viii^	2.5097 (11)
Ba1—Cu1^vi^	3.2778 (6)	Cu2—Cu4	2.5140 (9)
Ba1—Cu2^i^	3.5163 (7)	Cu2—Cu3^x^	2.5157 (9)
Ba1—Cu2^vii^	3.5163 (7)	Cu2—Cu4^x^	2.5404 (9)
Ba1—Cu2	3.5606 (7)	Cu3—Cu4^xii^	2.5633 (11)
Cu1—Cu2	2.5043 (9)	Cu3—Cu4^xiii^	2.7460 (5)
Cu1—Cu2^viii^	2.5043 (9)	Cu3—Cu4	2.7460 (5)
			
Cu1—Ba1—Cu4^i^	153.88 (2)	Cu3—Cu2—Ba1^i^	62.95 (2)
Cu1—Ba1—Cu3^ii^	76.80 (2)	Cu1—Cu2—Ba1^i^	124.28 (3)
Cu4^i^—Ba1—Cu3^ii^	77.08 (2)	Cu2^xi^—Cu2—Ba1^i^	69.096 (9)
Cu1—Ba1—Cu3^iii^	124.627 (16)	Cu2^viii^—Cu2—Ba1^i^	110.903 (9)
Cu4^i^—Ba1—Cu3^iii^	50.361 (13)	Cu4—Cu2—Ba1^i^	61.31 (2)
Cu3^ii^—Ba1—Cu3^iii^	81.212 (10)	Cu3^x^—Cu2—Ba1^i^	116.33 (2)
Cu1—Ba1—Cu3^i^	124.627 (16)	Cu1^vi^—Cu2—Ba1^i^	80.03 (2)
Cu4^i^—Ba1—Cu3^i^	50.361 (13)	Cu4^x^—Cu2—Ba1^i^	167.17 (3)
Cu3^ii^—Ba1—Cu3^i^	81.212 (10)	Cu3—Cu2—Ba1	80.12 (2)
Cu3^iii^—Ba1—Cu3^i^	100.71 (3)	Cu1—Cu2—Ba1	59.92 (2)
Cu1—Ba1—Cu4^iv^	81.686 (19)	Cu2^xi^—Cu2—Ba1	110.636 (9)
Cu4^i^—Ba1—Cu4^iv^	81.722 (10)	Cu2^viii^—Cu2—Ba1	69.365 (9)
Cu3^ii^—Ba1—Cu4^iv^	50.312 (12)	Cu4—Cu2—Ba1	125.65 (3)
Cu3^iii^—Ba1—Cu4^iv^	46.300 (19)	Cu3^x^—Cu2—Ba1	165.63 (3)
Cu3^i^—Ba1—Cu4^iv^	119.62 (2)	Cu1^vi^—Cu2—Ba1	62.497 (19)
Cu1—Ba1—Cu4^ii^	81.686 (19)	Cu4^x^—Cu2—Ba1	115.76 (2)
Cu4^i^—Ba1—Cu4^ii^	81.722 (10)	Ba1^i^—Cu2—Ba1	65.654 (13)
Cu3^ii^—Ba1—Cu4^ii^	50.312 (13)	Cu3—Cu2—Ba1^xiv^	59.59 (2)
Cu3^iii^—Ba1—Cu4^ii^	119.62 (2)	Cu1—Cu2—Ba1^xiv^	115.14 (2)
Cu3^i^—Ba1—Cu4^ii^	46.300 (19)	Cu2^xi^—Cu2—Ba1^xiv^	110.245 (9)
Cu4^iv^—Ba1—Cu4^ii^	100.62 (3)	Cu2^viii^—Cu2—Ba1^xiv^	69.755 (9)
Cu1—Ba1—Cu1^v^	49.969 (13)	Cu4—Cu2—Ba1^xiv^	61.011 (18)
Cu4^i^—Ba1—Cu1^v^	124.885 (15)	Cu3^x^—Cu2—Ba1^xiv^	60.956 (19)
Cu3^ii^—Ba1—Cu1^v^	80.84 (2)	Cu1^vi^—Cu2—Ba1^xiv^	163.78 (3)
Cu3^iii^—Ba1—Cu1^v^	76.838 (17)	Cu4^x^—Cu2—Ba1^xiv^	59.30 (2)
Cu3^i^—Ba1—Cu1^v^	162.05 (3)	Ba1^i^—Cu2—Ba1^xiv^	109.741 (17)
Cu4^iv^—Ba1—Cu1^v^	46.364 (19)	Ba1—Cu2—Ba1^xiv^	132.941 (16)
Cu4^ii^—Ba1—Cu1^v^	119.22 (2)	Cu3—Cu2—Ba1^vi^	164.61 (3)
Cu1—Ba1—Cu1^vi^	49.969 (13)	Cu1—Cu2—Ba1^vi^	61.115 (19)
Cu4^i^—Ba1—Cu1^vi^	124.885 (15)	Cu2^xi^—Cu2—Ba1^vi^	69.874 (9)
Cu3^ii^—Ba1—Cu1^vi^	80.84 (2)	Cu2^viii^—Cu2—Ba1^vi^	110.127 (9)
Cu3^iii^—Ba1—Cu1^vi^	162.05 (3)	Cu4—Cu2—Ba1^vi^	115.22 (2)
Cu3^i^—Ba1—Cu1^vi^	76.838 (17)	Cu3^x^—Cu2—Ba1^vi^	59.18 (2)
Cu4^iv^—Ba1—Cu1^vi^	119.22 (2)	Cu1^vi^—Cu2—Ba1^vi^	58.38 (2)
Cu4^ii^—Ba1—Cu1^vi^	46.364 (19)	Cu4^x^—Cu2—Ba1^vi^	60.531 (19)
Cu1^v^—Ba1—Cu1^vi^	99.92 (3)	Ba1^i^—Cu2—Ba1^vi^	132.043 (17)
Cu1—Ba1—Cu2^i^	155.461 (14)	Ba1—Cu2—Ba1^vi^	108.234 (16)
Cu4^i^—Ba1—Cu2^i^	43.679 (16)	Ba1^xiv^—Cu2—Ba1^vi^	106.895 (17)
Cu3^ii^—Ba1—Cu2^i^	114.50 (2)	Cu2^viii^—Cu3—Cu2	60.23 (3)
Cu3^iii^—Ba1—Cu2^i^	79.602 (18)	Cu2^viii^—Cu3—Cu2^xii^	156.30 (4)
Cu3^i^—Ba1—Cu2^i^	43.118 (17)	Cu2—Cu3—Cu2^xii^	114.536 (16)
Cu4^iv^—Ba1—Cu2^i^	122.57 (2)	Cu2^viii^—Cu3—Cu2^xv^	114.536 (16)
Cu4^ii^—Ba1—Cu2^i^	89.381 (18)	Cu2—Cu3—Cu2^xv^	156.30 (4)
Cu1^v^—Ba1—Cu2^i^	149.426 (17)	Cu2^xii^—Cu3—Cu2^xv^	59.83 (3)
Cu1^vi^—Ba1—Cu2^i^	108.340 (16)	Cu2^viii^—Cu3—Cu4^xii^	140.72 (3)
Cu1—Ba1—Cu2^vii^	155.461 (14)	Cu2—Cu3—Cu4^xii^	140.72 (3)
Cu4^i^—Ba1—Cu2^vii^	43.679 (16)	Cu2^xii^—Cu3—Cu4^xii^	59.33 (3)
Cu3^ii^—Ba1—Cu2^vii^	114.50 (2)	Cu2^xv^—Cu3—Cu4^xii^	59.33 (3)
Cu3^iii^—Ba1—Cu2^vii^	43.118 (17)	Cu2^viii^—Cu3—Cu4^xiii^	57.03 (2)
Cu3^i^—Ba1—Cu2^vii^	79.602 (18)	Cu2—Cu3—Cu4^xiii^	111.88 (3)
Cu4^iv^—Ba1—Cu2^vii^	89.381 (18)	Cu2^xii^—Cu3—Cu4^xiii^	112.01 (3)
Cu4^ii^—Ba1—Cu2^vii^	122.57 (2)	Cu2^xv^—Cu3—Cu4^xiii^	57.54 (2)
Cu1^v^—Ba1—Cu2^vii^	108.340 (16)	Cu4^xii^—Cu3—Cu4^xiii^	105.34 (2)
Cu1^vi^—Ba1—Cu2^vii^	149.426 (17)	Cu2^viii^—Cu3—Cu4	111.88 (3)
Cu2^i^—Ba1—Cu2^vii^	41.807 (19)	Cu2—Cu3—Cu4	57.03 (2)
Cu1—Ba1—Cu2	43.227 (16)	Cu2^xii^—Cu3—Cu4	57.54 (2)
Cu4^i^—Ba1—Cu2	155.890 (14)	Cu2^xv^—Cu3—Cu4	112.01 (3)
Cu3^ii^—Ba1—Cu2	113.91 (2)	Cu4^xii^—Cu3—Cu4	105.34 (2)
Cu3^iii^—Ba1—Cu2	148.851 (17)	Cu4^xiii^—Cu3—Cu4	132.09 (4)
Cu3^i^—Ba1—Cu2	108.245 (15)	Cu2^viii^—Cu3—Ba1^xiv^	77.99 (3)
Cu4^iv^—Ba1—Cu2	122.08 (2)	Cu2—Cu3—Ba1^xiv^	77.99 (3)
Cu4^ii^—Ba1—Cu2	89.354 (17)	Cu2^xii^—Cu3—Ba1^xiv^	78.32 (3)
Cu1^v^—Ba1—Cu2	78.967 (18)	Cu2^xv^—Cu3—Ba1^xiv^	78.32 (3)
Cu1^vi^—Ba1—Cu2	43.030 (17)	Cu4^xii^—Cu3—Ba1^xiv^	130.44 (4)
Cu2^i^—Ba1—Cu2	114.346 (13)	Cu4^xiii^—Cu3—Ba1^xiv^	66.05 (2)
Cu2^vii^—Ba1—Cu2	131.585 (13)	Cu4—Cu3—Ba1^xiv^	66.05 (2)
Cu2—Cu1—Cu2^viii^	60.14 (3)	Cu2^viii^—Cu3—Ba1^iii^	73.931 (19)
Cu2—Cu1—Cu2^ix^	155.73 (4)	Cu2—Cu3—Ba1^iii^	119.72 (3)
Cu2^viii^—Cu1—Cu2^ix^	114.397 (19)	Cu2^xii^—Cu3—Ba1^iii^	122.43 (3)
Cu2—Cu1—Cu2^vi^	114.397 (19)	Cu2^xv^—Cu3—Ba1^iii^	76.602 (19)
Cu2^viii^—Cu1—Cu2^vi^	155.73 (4)	Cu4^xii^—Cu3—Ba1^iii^	66.89 (2)
Cu2^ix^—Cu1—Cu2^vi^	59.67 (3)	Cu4^xiii^—Cu3—Ba1^iii^	63.582 (17)
Cu2—Cu1—Cu4^x^	60.01 (3)	Cu4—Cu3—Ba1^iii^	164.25 (3)
Cu2^viii^—Cu1—Cu4^x^	60.01 (3)	Ba1^xiv^—Cu3—Ba1^iii^	129.602 (13)
Cu2^ix^—Cu1—Cu4^x^	140.84 (3)	Cu2^viii^—Cu3—Ba1^i^	119.72 (3)
Cu2^vi^—Cu1—Cu4^x^	140.84 (3)	Cu2—Cu3—Ba1^i^	73.931 (19)
Cu2—Cu1—Cu1^vi^	57.51 (3)	Cu2^xii^—Cu3—Ba1^i^	76.602 (19)
Cu2^viii^—Cu1—Cu1^vi^	112.73 (5)	Cu2^xv^—Cu3—Ba1^i^	122.43 (3)
Cu2^ix^—Cu1—Cu1^vi^	111.77 (5)	Cu4^xii^—Cu3—Ba1^i^	66.89 (2)
Cu2^vi^—Cu1—Cu1^vi^	56.89 (3)	Cu4^xiii^—Cu3—Ba1^i^	164.25 (3)
Cu4^x^—Cu1—Cu1^vi^	104.97 (3)	Cu4—Cu3—Ba1^i^	63.583 (17)
Cu2—Cu1—Cu1^v^	112.73 (5)	Ba1^xiv^—Cu3—Ba1^i^	129.602 (13)
Cu2^viii^—Cu1—Cu1^v^	57.51 (3)	Ba1^iii^—Cu3—Ba1^i^	100.71 (3)
Cu2^ix^—Cu1—Cu1^v^	56.89 (3)	Cu2^xi^—Cu4—Cu2	59.87 (3)
Cu2^vi^—Cu1—Cu1^v^	111.77 (5)	Cu2^xi^—Cu4—Cu2^xvi^	113.220 (18)
Cu4^x^—Cu1—Cu1^v^	104.97 (3)	Cu2—Cu4—Cu2^xvi^	152.53 (4)
Cu1^vi^—Cu1—Cu1^v^	134.34 (7)	Cu2^xi^—Cu4—Cu2^xii^	152.53 (4)
Cu2—Cu1—Ba1	76.85 (3)	Cu2—Cu4—Cu2^xii^	113.220 (18)
Cu2^viii^—Cu1—Ba1	76.85 (3)	Cu2^xvi^—Cu4—Cu2^xii^	59.20 (3)
Cu2^ix^—Cu1—Ba1	78.89 (3)	Cu2^xi^—Cu4—Cu3^x^	59.39 (3)
Cu2^vi^—Cu1—Ba1	78.89 (3)	Cu2—Cu4—Cu3^x^	59.39 (3)
Cu4^x^—Cu1—Ba1	129.48 (4)	Cu2^xvi^—Cu4—Cu3^x^	143.57 (3)
Cu1^vi^—Cu1—Ba1	67.19 (3)	Cu2^xii^—Cu4—Cu3^x^	143.57 (3)
Cu1^v^—Cu1—Ba1	67.19 (3)	Cu2^xi^—Cu4—Cu1^xii^	143.74 (3)
Cu2—Cu1—Ba1^v^	122.72 (3)	Cu2—Cu4—Cu1^xii^	143.74 (3)
Cu2^viii^—Cu1—Ba1^v^	76.898 (19)	Cu2^xvi^—Cu4—Cu1^xii^	58.63 (3)
Cu2^ix^—Cu1—Ba1^v^	74.474 (19)	Cu2^xii^—Cu4—Cu1^xii^	58.63 (3)
Cu2^vi^—Cu1—Ba1^v^	119.61 (3)	Cu3^x^—Cu4—Cu1^xii^	104.50 (4)
Cu4^x^—Cu1—Ba1^v^	66.475 (19)	Cu2^xi^—Cu4—Cu3	111.18 (3)
Cu1^vi^—Cu1—Ba1^v^	162.71 (5)	Cu2—Cu4—Cu3	56.58 (2)
Cu1^v^—Cu1—Ba1^v^	62.84 (2)	Cu2^xvi^—Cu4—Cu3	110.69 (3)
Ba1—Cu1—Ba1^v^	130.031 (13)	Cu2^xii^—Cu4—Cu3	56.67 (2)
Cu2—Cu1—Ba1^vi^	76.898 (19)	Cu3^x^—Cu4—Cu3	104.61 (2)
Cu2^viii^—Cu1—Ba1^vi^	122.72 (3)	Cu1^xii^—Cu4—Cu3	104.18 (2)
Cu2^ix^—Cu1—Ba1^vi^	119.61 (3)	Cu2^xi^—Cu4—Cu3^xvii^	56.58 (2)
Cu2^vi^—Cu1—Ba1^vi^	74.474 (19)	Cu2—Cu4—Cu3^xvii^	111.18 (3)
Cu4^x^—Cu1—Ba1^vi^	66.475 (19)	Cu2^xvi^—Cu4—Cu3^xvii^	56.67 (2)
Cu1^vi^—Cu1—Ba1^vi^	62.84 (2)	Cu2^xii^—Cu4—Cu3^xvii^	110.70 (3)
Cu1^v^—Cu1—Ba1^vi^	162.71 (5)	Cu3^x^—Cu4—Cu3^xvii^	104.61 (2)
Ba1—Cu1—Ba1^vi^	130.031 (13)	Cu1^xii^—Cu4—Cu3^xvii^	104.18 (2)
Ba1^v^—Cu1—Ba1^vi^	99.92 (3)	Cu3—Cu4—Cu3^xvii^	132.09 (4)
Cu3—Cu2—Cu1	115.87 (3)	Cu2^xi^—Cu4—Ba1^i^	75.01 (2)
Cu3—Cu2—Cu2^xi^	120.112 (16)	Cu2—Cu4—Ba1^i^	75.01 (2)
Cu1—Cu2—Cu2^xi^	120.072 (16)	Cu2^xvi^—Cu4—Ba1^i^	77.54 (3)
Cu3—Cu2—Cu2^viii^	59.887 (16)	Cu2^xii^—Cu4—Ba1^i^	77.54 (3)
Cu1—Cu2—Cu2^viii^	59.929 (16)	Cu3^x^—Cu4—Ba1^i^	126.65 (4)
Cu2^xi^—Cu2—Cu2^viii^	180.0	Cu1^xii^—Cu4—Ba1^i^	128.85 (4)
Cu3—Cu2—Cu4	66.39 (2)	Cu3—Cu4—Ba1^i^	66.06 (2)
Cu1—Cu2—Cu4	174.38 (4)	Cu3^xvii^—Cu4—Ba1^i^	66.06 (2)
Cu2^xi^—Cu2—Cu4	60.062 (15)	Cu2^xi^—Cu4—Ba1^xviii^	76.585 (18)
Cu2^viii^—Cu2—Cu4	119.936 (15)	Cu2—Cu4—Ba1^xviii^	122.42 (3)
Cu3—Cu2—Cu3^x^	113.79 (2)	Cu2^xvi^—Cu4—Ba1^xviii^	76.763 (18)
Cu1—Cu2—Cu3^x^	113.52 (3)	Cu2^xii^—Cu4—Ba1^xviii^	122.09 (3)
Cu2^xi^—Cu2—Cu3^x^	60.085 (16)	Cu3^x^—Cu4—Ba1^xviii^	66.81 (2)
Cu2^viii^—Cu2—Cu3^x^	119.915 (16)	Cu1^xii^—Cu4—Ba1^xviii^	67.162 (19)
Cu4—Cu2—Cu3^x^	61.28 (3)	Cu3—Cu4—Ba1^xviii^	164.27 (3)
Cu3—Cu2—Cu1^vi^	135.92 (4)	Cu3^xvii^—Cu4—Ba1^xviii^	63.642 (17)
Cu1—Cu2—Cu1^vi^	65.604 (19)	Ba1^i^—Cu4—Ba1^xviii^	129.673 (13)
Cu2^xi^—Cu2—Cu1^vi^	60.164 (15)	Cu2^xi^—Cu4—Ba1^xiv^	122.42 (3)
Cu2^viii^—Cu2—Cu1^vi^	119.837 (15)	Cu2—Cu4—Ba1^xiv^	76.585 (18)
Cu4—Cu2—Cu1^vi^	116.79 (3)	Cu2^xvi^—Cu4—Ba1^xiv^	122.09 (3)
Cu3^x^—Cu2—Cu1^vi^	103.34 (3)	Cu2^xii^—Cu4—Ba1^xiv^	76.763 (19)
Cu3—Cu2—Cu4^x^	104.34 (3)	Cu3^x^—Cu4—Ba1^xiv^	66.81 (2)
Cu1—Cu2—Cu4^x^	61.36 (3)	Cu1^xii^—Cu4—Ba1^xiv^	67.162 (19)
Cu2^xi^—Cu2—Cu4^x^	119.601 (15)	Cu3—Cu4—Ba1^xiv^	63.642 (17)
Cu2^viii^—Cu2—Cu4^x^	60.399 (15)	Cu3^xvii^—Cu4—Ba1^xiv^	164.27 (3)
Cu4—Cu2—Cu4^x^	113.34 (2)	Ba1^i^—Cu4—Ba1^xiv^	129.673 (13)
Cu3^x^—Cu2—Cu4^x^	65.79 (2)	Ba1^xviii^—Cu4—Ba1^xiv^	100.62 (3)
Cu1^vi^—Cu2—Cu4^x^	112.24 (3)		

Symmetry codes: (i) −*x*+1, −*y*+1, −*z*; (ii) *x*+1/2, *y*, −*z*+1/2; (iii) −*x*+1, −*y*+2, −*z*; (iv) *x*+1/2, *y*+1, −*z*+1/2; (v) −*x*+1, −*y*+2, −*z*+1; (vi) −*x*+1, −*y*+1, −*z*+1; (vii) −*x*+1, *y*+1/2, −*z*; (viii) *x*, −*y*+3/2, *z*; (ix) −*x*+1, *y*+1/2, −*z*+1; (x) −*x*+1/2, −*y*+1, *z*+1/2; (xi) *x*, −*y*+1/2, *z*; (xii) −*x*+1/2, −*y*+1, *z*−1/2; (xiii) *x*, *y*+1, *z*; (xiv) *x*−1/2, *y*, −*z*+1/2; (xv) −*x*+1/2, *y*+1/2, *z*−1/2; (xvi) −*x*+1/2, *y*−1/2, *z*−1/2; (xvii) *x*, *y*−1, *z*; (xviii) *x*−1/2, *y*−1, −*z*+1/2.
